# Draft Genome Sequence of *Pseudoalteromonas* sp. Strain R3, a Red-Pigmented l-Amino Acid Oxidase-Producing Bacterium

**DOI:** 10.1128/genomeA.01339-15

**Published:** 2015-11-19

**Authors:** Zhiliang Yu, Minyan Zhao, Juanping Qiu

**Affiliations:** College of Biological and Environmental Engineering, Zhejiang University of Technology, Hangzhou, China

## Abstract

Here, we report a draft 5.58-Mb genome sequence of *Pseudoalteromonas* sp. strain R3, isolated from an intertidal-zone sludge sample, which has l-amino acid oxidase activity. The genomics information of this strain will facilitate the study of l-amino acid oxidase, quorum sensing, and the relationship of the two.

## GENOME ANNOUNCEMENT

Members of the genus *Pseudoalteromonas* (*Gammaproteobacteria*, *Alteromonadales*, *Pseudoalteromonadaceae*) have been isolated from different niches around the globe, including seawater (1), sediment (2), sea ice (3), and tidal flats (4). *Pseudoalteromonas* is increasingly recognized as ecologically and biologically important microbe due to its influence on biofilms and its ability to synthesize bioactive molecules (5). *Pseudoalteromonas* sp. strain R3 was isolated from an intertidal-zone sludge sample (30.03°N, 122.11°E) located at Dinghai, Zhoushan, China, and it has been found to have l-amino acid oxidase activity, which can catalyze the oxidative deamination of l-amino acids to the corresponding α-keto acids with a release of ammonia and hydrogen peroxide (6). *Pseudoalteromonas* sp. R3 can also produce a red pigment and form a biofilm, which is probably regulated by quorum sensing (7). We sequenced its whole genome in order to find out whether the l-amino acid oxidase activity is regulated by quorum sensing.

The genome of *Pseudoalteromonas* sp. R3 was sequenced with an Illumina HiSeq 2500 platform by Nextomics Biosciences Co., Ltd. (Wuhan, China). A paired-end library with a fragment length of 300 to 500 bp, generating a total of 17,346,435 high-quality paired reads, with an average coverage of approximately 364.1×, was used. The genome was assembled by SOAP*denovo* version 2.04 (http://soap.genomics.org.cn/) with multiple *k*-mer parameters. GapCloser version 1.12 software was subsequently applied to fill the remaining local inner gaps and correct the single-base polymorphism for the final assembly result. Glimmer version 3.02 (http://www.cbcb.umd.edu/software/glimmer/) was used to predict protein-coding open reading frames (ORFs). For RNA predictions, tRNAs were predicted by tRNAscan-SE version 1.3.1 (8), and rRNAs were predicted by RNAmmer-1.2 (9). The NCBI nonredundant (NR), Swiss-Prot (http://Uniprot.org), KEGG (http://www.genome.jp/kegg), and Clusters of Orthologous Group (COG) (http://www.ncbi.nlm.nih.gov/COG) databases were used for functional annotation.

The draft genome is estimated to 5.58 Mb, with a G+C content of 47.63% and 5,429 putative ORFs, with an average size of 944 bp. Approximately 87.8% of nucleotides were predicted to be ORFs. Furthermore, strain R3 contains 13 rRNA operons and a total of 115 tRNA genes. The genome analysis confirms the presence of the l-amino acid oxidase gene and genes related to quorum sensing.

### Nucleotide sequence accession numbers.

The *Pseudoalteromonas* sp. R3 whole-genome shotgun project has been deposited at DDBJ/EMBL/GenBank under the accession number LJDF00000000. The version described in this paper is the first version, LJDF01000000.
